# The intake of monosodium aspartate attenuates aggression induced by post-weaning social isolation in an ADHD rat model

**DOI:** 10.1016/j.jphyss.2026.100056

**Published:** 2026-01-09

**Authors:** Yu Nishimura, Dewi Mustika, Shinya Ueno, Shiori Tominaga, Mariko Shindo, Naoki Tajiri, Cha-Gyun Jung, Hideki Hida

**Affiliations:** a Department of Neurophysiology and Brain Science, Nagoya City University Graduate School of Medical Science, Mizuho-ku, Nagoya 467-8601, Japan; b Department of Physiology, Faculty of Medicine, Universitas Brawijaya, Malang, Indonesia

**Keywords:** Umami, Monosodium aspartate, C-Fos, Aggression, Gut-brain axis, Vagus nerve

## Abstract

We investigated whether monosodium aspartate (MSA), an umami compound structurally analogous to monosodium glutamate (MSG), influences aggressive behavior in a rat model of attention-deficit/hyperactivity disorder. Spontaneously hypertensive rats (SHR/Izm) were subjected to post-weaning social isolation and assessed using the resident-intruder paradigm. MSA ingestion significantly reduced aggression, particularly the frequency and duration of attacks, while the open field test showed no differences in anxiety-like behavior. c-Fos immunohistochemistry revealed increased neuronal activation in the intermediate nucleus of the solitary tract (iNTS) and decreased activation in the central amygdala (CeA) following MSA ingestion. This effect, along with the reduction in aggression, was abolished by vagotomy, suggesting gut-brain involvement. These findings indicate that MSA, like MSG, can reduce aggression via the gut-brain axis, implicating the vagus nerve, iNTS and CeA as key mediators. This highlights that the modulation of aggression by ingested amino acids is a broader effect acting through shared mechanisms.

## Highlights

• MSA reduces aggression in ADHD rat model via gut-brain interaction.
• MSA activates iNTS and modulate CeA similarly to MSG, confirmed by c-Fos expression.
• Vagotomy blocks iNTS activation, CeA modulation and MSA's anti-aggressive effects.
• MSA does not alter anxiety-like behavior in open field test.
• MSA may be a safer alternative to MSG for behavioral modulation.

## 1 Introduction

Attention-deficit/hyperactivity disorder (ADHD) is a neurodevelopmental disorder characterized by symptoms of inattention, hyperactivity, and impulsivity, typically manifesting before preschool age. It is frequently associated with aggressive behaviors [1], [2], [3]. Empirical studies have demonstrated that children experiencing social isolation, such as neglect or insufficient peer interaction, exhibit increased aggression and a heightened risk of psychiatric disorders [4], [5]. Furthermore, variations in the external environment during developmental periods significantly influence later life outcomes [6], [7], [8]. These findings underscore the critical importance of early environmental interventions in mitigating these risks. Consequently, early intervention and supportive environments can substantially enhance symptom management and improve long-term outcomes [9], [10], [11].

From an environmental intervention perspective, nutrition by food during the developmental period is of paramount importance. Food consists of five fundamental taste components: sweet, salty, sour, bitter, and umami. Notably, umami perception is believed to develop from the neonatal period, as breast milk contains abundant glutamate [12] an umami substance that impacts a "pleasant" postprandial sensation. Studies have shown that infants given a umami substance, monosodium glutamate (MSG), exhibit a calm expression, in contrast to the doubtful reaction observed when given caffeine, a bitter-tasting substance [13], [14]. Interestingly, research on gustatory preferences indicates that a proclivity for bitter and spicy foods is associated with elevated levels of aggression and specific personality traits, including hostility and psychopathy [15], [16], [17], [18], [19]. The findings suggest a significant interconnection between taste perception, eating behaviors, and emotional states.

Recognizing the significance of early environmental interventions for ADHD, it is essential to understand the relationship between taste stimulation and emotional development. Our recent study has identified umami taste as an external stimulus that affects emotional regulation: the consumption of MSG during the developmental period reduces aggression in an animal model of ADHD. This effect is mediated through signal transduction from the upper gastrointestinal tract to the nucleus of the solitary tract (NTS) in the medulla oblongata via the vagus nerve. Our findings highlight the substantial impact of early environmental factors on emotional development and underscore the importance of non-pharmacological supportive approaches in the management of ADHD [20], [21].

Nonetheless, it remains uncertain whether these effects are exclusive to monosodium glutamate (MSG) or if other umami compounds exert similar influences. Monosodium aspartate (MSA) possesses a molecular structure similar to that of MSG, comprising an amino acid group and an ionic sodium component, which enables it to interact with the same taste receptors, particularly the umami receptors (T1R1/T1R3) [22], [23]. Thus, MSA may potentially exhibit comparable effects on aggression and symptoms akin to attention-deficit/hyperactivity disorder (ADHD). The present study aimed to examine whether the ingestion of MSA produces effects on aggression and ADHD-like symptoms comparable to those observed during the developmental period.

## 2 Methods

### 2.1 Animals

The Nagoya City University Medical School Animal Experimental Committee granted approval for all animal experiments, which were conducted in full compliance with established animal care guidelines. Considerable efforts were made to limit the number of animals used and to mitigate any potential suffering.

**Fig. 1:**
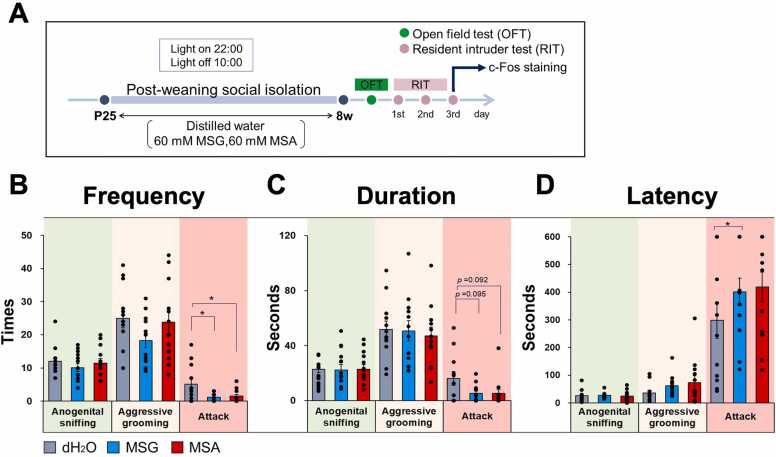
The ingestion of MSA resulted in a reduction of PWSI-induced aggressive behavior, as demonstrated by the resident-intruder test. (A)　This figure presents a schematic representation of the experimental protocol. The resident-intruder test was administered during the subjective night following a five-week housing period. (B, C) MSA ingestion led to a decrease in both the frequency and duration of attack behavior. (D) No significant difference was observed in the latency of aggressive behavior between the MSA and control groups. Data are presented as the mean ± SE (n = 12 in each group); *p < 0.05, **p < 0.01, one-way ANOVA followed by Tukey’s test.

In brief, spontaneously Hypertensive Rats (SHR/Izm) (SLC Inc, Hamamatsu, Japan), recognized as a model for aggressive ADHD, were utilized in this study [21], [24]. The rats were maintained under a 12-hour light/dark cycle, with lights on at 22:00 and off at 10:00, in a temperature-controlled environment set between 23–25 °C and an average humidity of 50 %. They were provided with chow (MFG; Oriental Yeast Co. Ltd.) and one of the following solutions ad libitum: distilled water (dH2O), 60 mM MSG (MP Biomedicals, USA, 101800), or 60 mM MSA (FUJIFILM Wako Pure Chemical Corp., Osaka, Japan) (Fig. 1A).

Post-weaning social isolation (PWSI) was conducted on rats that were individually housed in cages measuring 40 × 23 × 18 cm, with minimal sawdust, from post-weaning at P25 until reaching maturity at P60, over a duration of five weeks, to induce stable aggression. Behavioral assessments were conducted during the dark phase, specifically between 13:00 and 16:00. In the resident-intruder paradigm, Wistar/ST rats were utilized as intruders and were maintained in enclosures with a 12-hour light/dark cycle, with lights activated at 8:00 and deactivated at 10:00. The intruders were provided with ad libitum access to food and water.

### 2.2 Behavioral tests

#### 2.2.1 Resident intruder test

The Resident Intruder Test was conducted during the dark phase to evaluate aggression in SHR/Izm rats. A male Wistar/ST rat, approximately 20 g lighter than the resident, was introduced into the resident's home cage and designated as the intruder. The resident, an SHR/Izm rat, was observed for aggressive responses upon the introduction of the intruder. Behavioral recordings were performed under dim red light (<2 lx) between 13:00 and 16:00 using a video camera [25]. The test was conducted over three consecutive days using the same resident-intruder pair to ensure consistency. Behavioral data from the second day were analyzed, as aggression levels were more stable at this time point. Aggressive behaviors were classified into three categories based on previously established criteria: anogenital sniffing (weak aggression), allo-grooming (moderate aggression), characterized by mounting and pouncing, and attack (strong aggression), characterized by biting and keeping down [26]. Each aggression category was analyzed for frequency, duration, and latency over a 10-minute observation period. Behavioral assessments were manually conducted using the Smart software (Bio Research Center Inc., Nagoya, Japan).

#### 2.2.2 Open field test

The open field test was conducted during the dark phase prior to the resident intruder test (P60). This assessment aimed to evaluate anxiety-like behavior in a novel environment over a 10-min period. Rats were positioned at the center of a black circular arena (60 cm in diameter × 50 cm in height) during the dark phase, between 13:00 and 16:00, under red dim light (<2 lx). Anxiety-like behavior was assessed using three indicators: total distance traveled, number of entries into the center area, and duration spent in the center area over the 10-min period [27], [28]. Behaviors recorded via a video camera were analyzed using the Smart software automatic tracking system (Bio Research Center Inc., Nagoya, Japan).

### 2.3 Immunohistochemistry of c-Fos

To evaluate the neuronal activity associated with taste stimulation following MSA ingestion, c-Fos immunohistochemistry was conducted [29], [30]. Ninety minutes subsequent to each experimental condition (resident intruder test on day 3 or intragastric administration test), the animals were deeply anesthetized with an intraperitoneal injection of sodium pentobarbital (100 mg/kg; Tokyo Kasei, Tokyo, Japan) and transcardially perfused with 0.1 M phosphate-buffered saline (PBS, pH 7.4), followed by 4 % (w/v) paraformaldehyde (PFA, Sigma Aldrich, St. Louis, USA) in PBS for fixation. The brains were obtained and post-fixed overnight in 4 % PFA and subsequently cryoprotected in a 30 % (w/v) sucrose solution at 4 °C. Brain samples were embedded in O.C.T. compound (Sakura Finetek Japan Co., Ltd.), sectioned coronally at a thickness of 40 μm using a cryostat (Leica CM1520, Japan), and stored in an antifreeze solution containing 25 % ethylene glycol and 25 % glycerol in PB solution.

The brain sections were rinsed in PBS (3 × 5 min) and subsequently incubated in PBS containing 20 % methanol and 3 % H₂O₂ (Wako, Tokyo, Japan) for 30 min to inactivate endogenous peroxidase. Following additional PBS washes, non-specific binding was blocked using 10 % horse serum in PBS with 0.1 % Triton X-100 (PBST, Nacalai Tesque, Inc., Kyoto, Japan) for 60 min, followed by rinsing with PBST. The sections were then incubated overnight at 4 °C with a mouse monoclonal anti-c-Fos antibody (EnCor Biotechnology Inc., Florida, MCA-2H2, lot #011024) diluted 1:1000 in PBST containing 1 % normal horse serum (Vector Laboratories, Inc., California, S-2000, lot #ZJ0329).

On the second day, the sections were rinsed in PBST containing 1 % horse serum and incubated for 120 min at room temperature (RT) with biotinylated anti-mouse IgG (Vector Laboratories, Inc., California, BA-2000, lot #ZB0622) diluted 1:200 in PBST with 1 % normal horse serum. Following PBS washes, the sections were incubated for 60 min at RT with an avidin-biotin complex (Vectastain ABC Kit, Vector Laboratories, Inc., USA, PK-4000, lot #ZK0713) diluted 9 µL/mL in PBST containing 1 % normal horse serum. Immunoreactivity was visualized on ice using 0.25 mg/mL diaminobenzidine (DAB) in PBS with 0.3 μL/mL of 30 % H₂O₂.The sections were subsequently mounted on gelatin-coated slides, dehydrated through a graded ethanol series (50–100 %), and sealed. The samples were examined using a light microscope, and images were captured in accordance with the brain atlas. c-Fos-positive cells (c-Fos+ cells) were manually quantified utilizing ImageJ software.

### 2.4 Vagotomy

To elucidate the pathway through which MSA ingestion exerts its effects, a vagotomy was performed on male SHR/Izm rats at P24 [31]. The animals were anesthetized intraperitoneally with a mixed solution at a dosage of 2 mL/kg, comprising medetomidine (0.185 mg/mL; Fujita Pharmaceutical Co., Ltd., Tokyo, Japan), midazolam (1 mg/mL; Alfresa Holdings Co., Ltd., Tokyo, Japan), and butorphanol (1.25 mg/mL; Meiji Animal Health Co., Ltd., Tokyo, Japan). Following a median incision below the diaphragm, the liver was protected using saline-soaked gauze, and the anterior and posterior vagal trunks adjacent to the lower esophagus were severed. The organ was then repositioned appropriately, and the muscle layer and skin were sutured sequentially. Postoperatively, the rats received an intraperitoneal administration of atipamezole (0.235 mg/mL; Nippon Zenyaku Kogyo Co., Ltd., Fukushima, Japan) at a dosage of 1 mL/kg. The day following surgery, the rats were individually housed and provided with MSA via a drinking bottle for a duration of five weeks until the commencement of behavioral tests.

### 2.5 Statistical analysis

Statistical analyses were executed using the EZR software, with a threshold for statistical significance set at p ≤ 0.05. The data are reported as mean ± standard error of the mean (SEM). In the resident intruder test, the frequency and duration metrics were examined using one-way ANOVA, followed by Tukey’s post hoc test. Latency was assessed through non-parametric analysis, followed by the Steel test, due to the endpoint being established at 10 min. In the vagotomy experiment, aggression-related data, excluding latency, were subjected to one-way ANOVA, with Dunnett’s post hoc test applied subsequently. Latency was analyzed independently using non-parametric methods, followed by the Steel test. The open field test and c-Fos immunohistochemistry (IHC) data were both analyzed using one-way ANOVA, followed by Tukey’s post hoc test.

## 3 Results

### 3.1 MSA ingestion reduced PWSI-induced aggression in SHR/Izm rats

Our previous studies have demonstrated that the oral administration of MSG during developmental stages reduces aggressive behavior induced by PWSI [32]. However, it remains unclear whether this effect is unique to MSG. To investigate this, we assessed the effects of MSA, a structurally similar compound, when administered orally during development.

To evaluate aggression, the resident intruder test was administered, and behavioral data from day 2 were analyzed (Fig. 1A). Similar to the MSG group, the ingestion of MSA significantly decreased the frequency of attacks (indicative of strong aggression) and exhibited a declining trend in attack duration compared to the control group (Fig. 1B, C). However, no significant differences were observed in anogenital sniffing (indicative of weak aggression), aggressive grooming (indicative of moderate aggression), or attack latency (Fig. 1D). Furthermore, no significant differences were identified between the MSG and MSA groups in overall aggression (Fig. 1B-D).

**Fig. 2:**
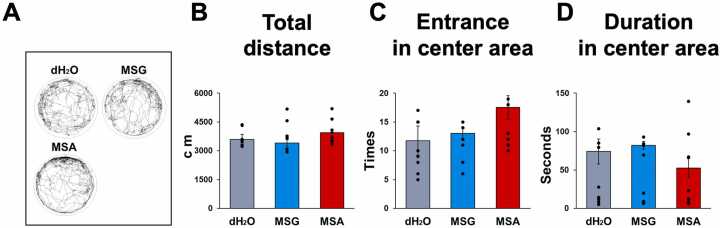
The ingestion of MSA did not influence the anxiety-like behaviors assessed by the Open Field Test. (A) Representative track plot reports were documented during 10-minute test sessions. (B-D) No statistically significant differences were identified in the total distance traveled, the number of entries into the center, or the duration spent in the center area between the groups. Data are expressed as the mean ± SE (n = 12 in each group); *p < 0.05, **p < 0.01, one-way ANOVA followed by Tukey’s test.

To assess anxiety-like behavior, the open field test was conducted following five weeks of PWSI. Measurements of total distance traveled, center entries, and center duration indicated no significant differences between the groups (Fig. 2A-D).

### 3.2 The number of c-Fos-positive cells significantly increased in the iNTS and decreased in the CeA by MSA ingestion

**Fig. 3:**
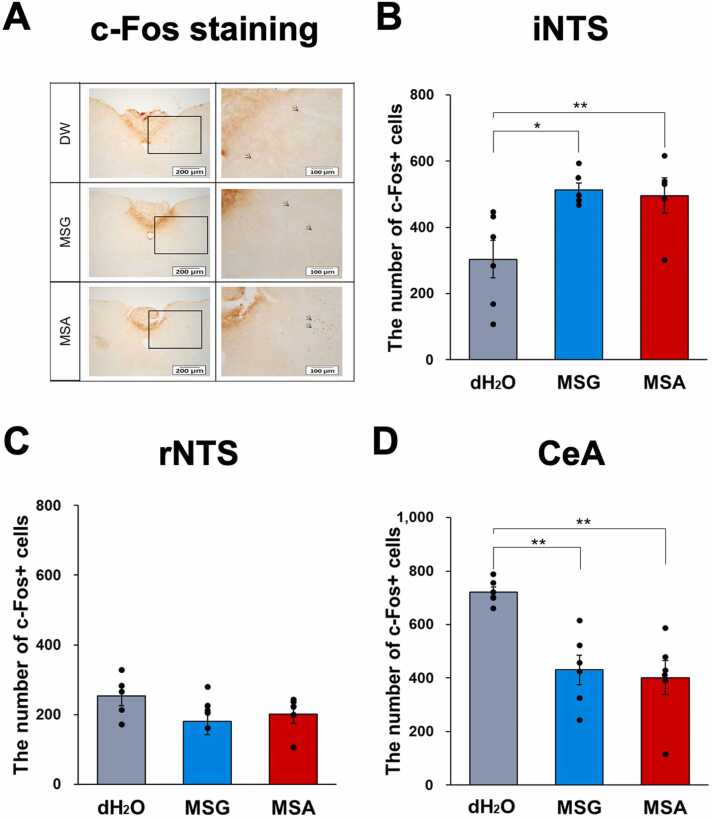
The impact of MSA ingestion on the quantity of c-Fos+ cells within the nucleus of the solitary tract and the central amygdala. (A) Photomicrographs of c-Fos-positive cells in the iNTS. (B) The quantity of c-Fos+ cells within the iNTS exhibited a significant increase subsequent to the ingestion of MSA and MSG. (C) No difference in c-Fos+ cells was observed in the rNTS for all groups. (D) The quantity of c-Fos+ cells within the CeA exhibited a significant decrease subsequent to the ingestion of MSA and MSG. Data are expressed as the mean ± SE (n = 12 in each group); *p < 0.05, **p < 0.01, Kruskal Wallis followed by Uncorrected Dunn Test.

To determine whether the observed reduction in aggression following MSA ingestion is mediated by taste perception in the tongue or the gut, c-Fos immunostaining was performed in the NTS. This region is crucial for taste signal transduction from both the tongue (rostral part of NTS/rNTS) and the small intestine (intermediate part of NTS/iNTS) [33], [34]. The MSA group, similar to the MSG group, exhibited a significant increase in c-Fos+ cells in the iNTS compared to the control group. However, no significant differences were observed in c-Fos+ cell counts in the rNTS among the groups (Fig. 3A-C).

In contrast, within the central amygdala (CeA)—a key brain region involved in the regulation of emotional and aggressive behaviors—the number of c-Fos–positive neurons significantly decreased in both the MSA and MSG groups compared to controls (Fig. 3D).

### 3.3 Neuronal activity within the iNTS is significantly increased following the intragastric administration of MSA

**Fig. 4:**
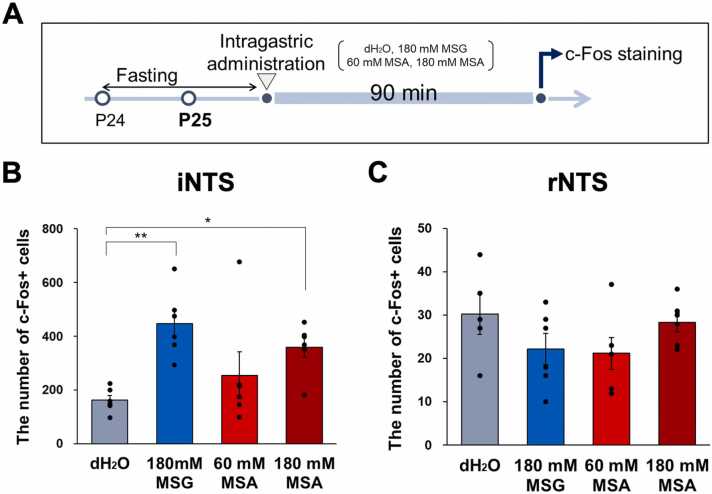
The intragastric administration results in an increased expression of c-Fos in the iNTS. (A) The experimental protocol is schematically illustrated as follows: Following an overnight fasting period, the rats were administered one of the following solutions via intragastric administration: dH2O, 180 mM MSG, 60 mM MSA, or 180 mM MSA. (B-C) The administration of 180 mM MSA resulted in a significant increase in c-Fos+ cells within the iNTS, comparable to the effects observed with 180 mM MSG. Data are expressed as the mean ± SE (n = 5–6); *p < 0.05, Kruskall Wallis followed by Uncorrected Dunn Test.

To verify direct activation via the gastrointestinal tract, P24 male SHR/Izm rats were subjected to an overnight fast and subsequently administered 0.75 mL intragastric infusions of dH2O, 180 mM MSG, 60 mM MSA, or 180 mM MSA solution. After a period of 90 min, the brains were perfused and stained for c-Fos in the rNTS and iNTS, followed by an assessment of the number of c-Fos+ cells (Fig. 4A).

The findings indicated a significant increase in c-Fos+ cells in the iNTS following the administration of 180 mM MSG and 180 mM MSA, whereas no significant differences were observed in the rNTS across the groups (Fig. 4B, C).

### 3.4 The reduction in aggression resulting from MSA ingestion is facilitated by the vagus nerve

**Fig. 5:**
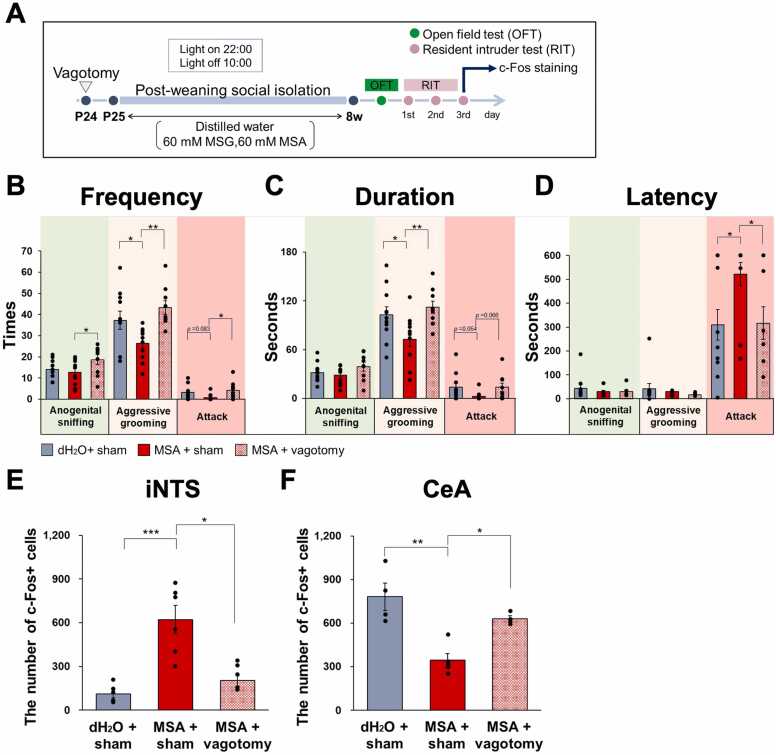
The role of the vagus nerve in mitigating aggression following MSA consumption. (A) Schematic illustration of the experimental protocol. (B-C) Vagotomy effectively counteracted the decrease in both the frequency and duration of aggressive grooming and attack behaviors that were induced by MSA ingestion, thereby restoring aggression levels to those comparable to the dH2O ingestion group. (D) The latency of attack was markedly reduced in the MSA + vagotomy group. (E) The expression of c-Fos in the iNTS was markedly diminished in the MSA + vagotomy group compared to the MSA + sham group. (F) the MSA-induced reduction in c-Fos–positive neurons were blocked by vagotomy, showing a significance increasing in MSA + vagotomy group compared with MSA + sham group. Data are expressed as mean ± SE (n = 9–11 for the behavioral experiment, n = 4–6 for staining); *p < 0.05, **p < 0.01, one-way ANOVA followed by Dunnett's test (for the behavioral experiment) and Kruskal Wallis followed by Uncorrected Dunn Test (for the c-Fos experiment).

To evaluate the role of the vagus nerve in mediating the aggression-suppressing effects of MSA ingestion, aggressive behaviors were compared between sham-operated (sham) and vagotomized (vagotomy) groups following a five-week period of individual housing and MSA ingestion (Fig. 5A).

In the group treated with MSA and subjected to a sham operation, there were observed reductions in both the frequency and duration of aggressive grooming and attacks. Conversely, in the MSA-treated group that underwent vagotomy, significant reductions in these parameters were noted, accompanied by a trend towards increased attack duration. Additionally, the extended latency of attack observed in the MSA-treated sham group was diminished following vagotomy (Fig. 5B-D).

The analysis of c-Fos immunostaining revealed that the increase in c-Fos+ cells within the iNTS, induced by MSA, was nullified in the group subjected to both MSA and vagotomy (Fig. 5E). A similar effect was observed in the CeA, where the MSA-induced reduction in c-Fos–positive neurons was also blocked by vagotomy, showing a significance increasing in MSA + vagotomy group compared with MSA + sham group (Fig. 5F).

## 4 Discussion

This study examined whether the ingestion of MSA, an umami compound chemically akin to MSG, during the developmental phase, exerts effects on aggression in SHR/Izm rats, a widely acknowledged animal model for ADHD, similar to those of MSG through an identical mechanism. SHR/Izm rats do not typically exhibit stable aggressive behavior under standard group-housing conditions, as confirmed by our preliminary observations (Supplementary Figure S1). Therefore, post-weaning social isolation (PWSI) was employed to reliably escalate aggression in this strain. Even though PWSI generally increases aggression in male rodents, the intensity and characteristics of this behavior are highly strain-dependent [35]. This approach is consistent with previous reports showing that PWSI enhances aggression in male rodents, with SHR/Izm rats displaying more robust aggressive responses than their genetic controls, such as WKY rats, or others rats strain [21].

We acknowledge that the behavioral effects of MSG observed in this study were somewhat weaker than those reported previously, and that the magnitude of MSA’s effects differed between experiments [21]. Such variability may arise from differences in baseline aggression levels across SHR/Izm cohorts subjected to PWSI, a factor known to fluctuate naturally even under controlled housing and testing conditions [36], [37]. Differences in sample size and subtle environmental factors may have also contributed to these variations [38]. Despite this variability, the overall pattern across experiments remained consistent: both MSG and MSA reduced aggressive behavior relative to controls. Future studies with larger cohorts and refined behavioral controls will be important to enhance reproducibility and to more precisely identify the biological and environmental factors contributing to these differences.

Using this model, the resident intruder test demonstrated that both the MSG- and MSA-treated groups exhibited a significant reduction in aggressive behavior compared to the control group. No significant differences were observed between the MSG and MSA groups, suggesting that MSA mitigates PWSI-induced aggression to a degree comparable to MSG. These findings imply that the stimulation of umami receptors, irrespective of the specific umami compound, may exert similar modulatory effects on behavior, particularly in terms of aggression. While SHR/Izm rats provide a robust platform for investigating ADHD-related aggression, it remains to be determined whether the anti-aggressive effects of MSG and MSA observed here are unique to this strain or generalizable to other rodent models under social isolation stress. Further comparative studies across strains will be essential to clarify the strain specificity and broader translational relevance of these findings.

Given these behavioral findings, it is important to consider the underlying mechanisms that may account for the observed anti-aggressive effects. However, these effects may also reflect the general neurochemical properties of glutamate and aspartate as amino acids, rather than being exclusively attributable to umami receptor activation. Although both MSG and MSA elicit umami taste in humans, previous studies have shown that rodent T1R1/T1R3 receptors are less sensitive to glutamate and aspartate compared with their human counterparts, and that other amino acids such as alanine and serine more effectively activate rodent umami receptors [39]. For this reason, the concentrations of MSG and MSA used in this study were approximately 20-fold higher than the estimated human daily intake to achieve sufficient receptor stimulation in rodents (Supplementary Figure 2) [39], [40]. In this study, the human equivalent dose (HED) was estimated from the daily intake per kilogram body weight (kgBW/d) of rats, involving allometric scaling by multiplying with the Km ratio of 0.162 and adjusting for the low sensitivity of the taste receptor T1R1/T1R3 in rodents by dividing by 20 [39], [41]. Based on these data, the MSG and MSA daily intake in this experiment is estimated to be equal to 20.119–25.316 mg/kgBW/d and 23.016–26.933 mg/kgBW/d in humans (Supplementary Figure S2). Further studies comparing the effects of other amino acids capable of activating T1R1/T1R3 would help clarify whether the observed behavioral changes are specific to umami–mediated receptor signaling or represent broader amino acid–mediated effects.

The capacity of monosodium glutamate (MSG) and monosodium aspartate (MSA) to engage with the same receptor system may lead to similar behavioral modulation through gut-brain communication via vagal pathways [42], [43]. Despite the distinct molecular structures of MSA and MSG, both compounds activate umami taste receptors, specifically G protein-coupled receptors such as T1R1/T1R3, mGlu1, and mGlu4, which are expressed in the lumen of the gastrointestinal tract [44], [45]. Previous studies have demonstrated that L-glutamate can activate T1R1/T1R3 independently at physiologically relevant concentrations, while L-aspartate requires the presence of 5’-ribonucleotides such as IMP or GMP for effective activation [40], [46]. This difference likely reflects a weaker ligand binding within the receptor’s Venus flytrap domain due to aspartate’s shorter side chain, resulting in lower conformational stability and efficacy [40], [47]. In our study, both MSG and MSA increased neuronal activity in the intermediate nucleus of the solitary tract (iNTS) and decreased activity in the central amygdala (CeA), suggesting engagement of a shared gut–brain signaling mechanism. The slightly weaker behavioral effect of MSA compared with MSG may therefore stem from its lower receptor potency or its dependence on luminal nucleotide cofactors for effective receptor activation. Future studies are warranted to further characterize receptor binding dynamics and delineate the specific neural pathways mediating this anti-aggressive effect.

Interestingly, although MSG and MSA were administered orally, we observed increased c-Fos expression in the iNTS but not in the rNTS. This discrepancy may be explained by differences in temporal dynamics and the relative strength of oral versus post-ingestive inputs. Gustatory afferents projecting to the rNTS are typically phasic, activated only during licking, and produce transient neural responses that may not be detectable 90 min after behavioral testing in this study [48], [49]. In contrast, gut-derived vagal afferents targeting the iNTS generate more sustained activity following nutrient ingestion, which aligns better with the c-Fos detection window [50], [51], [52]. Another possibility is that oral responses to MSG and MSA were masked by the complex tastants present in standard chow, thereby reducing the likelihood of detecting differences in rNTS activation between experimental and control groups. Together, these factors suggest that the iNTS activation observed here predominantly reflects post-ingestive vagal signaling rather than transient orosensory input.

The signals triggered by both MSG and MSA ascend via vagal afferents to the intermediate nucleus of the solitary tract (iNTS), which projects directly or indirectly to the central amygdala (CeA)—a key region for modulating emotional, defensive, and aggressive behaviors [21], [53]. In this study, ingestion of MSG and MSA led to increased neuronal activity in the iNTS accompanied by a concurrent decrease in c-Fos expression in the CeA specifically under conditions of PWSI-induced CeA hyperactivity. This inverse activation pattern suggests a functional coupling between vagal afferent stimulation and decreasing of pathological CeA hyperexcitability [21]. The CeA serves as an integrative hub that receives excitatory input from sensory and limbic areas to drive reactive aggression [54], [55], [56]. Therefore, the attenuated neuronal hyperactivity within this region following MSG or MSA ingestion may reflect enhanced inhibitory GABAergic signaling or decreased excitatory glutamatergic drive, potentially mediated through iNTS–amygdala projection. Consistent with this interpretation, previous studies have reported that activation of the vagus nerve or other gut–brain pathways diminish CeA excitability and reduce aggressive or anxiety-like behaviors [21], [57], [58]. Together, these findings support the hypothesis that umami-induced activation of the gut–brain axis engages inhibitory neural circuits that attenuate pathological CeA output, thereby mitigating aggression. Future studies should investigate whether this CeA modulation involves specific neurotransmitter systems—particularly GABAergic inhibition, noradrenergic modulation, and serotonergic signaling—to clarify the neurochemical mechanisms underlying umami-mediated behavioral regulation.

The reduction in aggression may also be mediated by the modulation of stress and anxiety conditions. Given that PWSI is known to induce heightened reactive aggression, often triggered by emotions such as frustration, anxiety, and fear, the open field test was utilized to determine whether umami modulation of aggression occurs through a reduction in anxiety. The results revealed no significant differences between groups, indicating that MSA ingestion did not produce an anxiolytic effect. This suggests that the observed reduction in aggressive behavior associated with MSA ingestion is not due to decreased anxiety but rather to a distinct neurobiological mechanism. These findings are consistent with previous studies in MSG effect, further supporting the hypothesis that the aggression-suppressing effect of umami stimulation operates independently of anxiety modulation [21].

While both glutamate and aspartate serve as excitatory neurotransmitters within the central nervous system (CNS), their roles in excitotoxicity differ [59]. Although aspartate can activate NMDA receptors, it is less prevalent in the CNS and exhibits a lower affinity for these receptors compared to glutamate [60]. This reduced prevalence and receptor affinity make aspartate less likely to induce excessive receptor activation, thereby reducing the risk of excitotoxicity. Unlike MSG, which contains glutamate, MSA comprises aspartate, an amino acid that also activates umami taste receptors (T1R1/T1R3) but follows a distinct metabolic pathway [61]. MSA may represent a potentially safer and more versatile alternative to MSG, particularly in relation to glutamate sensitivity and neurophysiological effects. Thus, MSA's ability to activate the same umami receptors suggests that it can offer a similar taste enhancement effect to MSG while potentially reducing the risk of glutamate overexposure.

Given its potential to modulate aggression and emotional regulation with reduced excitatory risks, MSA may serve as a valuable alternative in functional food formulations targeting behavioral health. Future research should further explore its long-term safety profile and its application in dietary interventions aimed at mitigating glutamate-related sensitivities.

## 5 Conclusion

In conclusion, our findings demonstrate that MSA exerts an effect comparable to MSG in mitigating aggressive behavior in an ADHD rat model. This suggests that the modulation of aggression by umami is not exclusive to MSG but is possibly mediated through the activation of shared receptors; however further study is needed to confirm. Other umami substances, such as MSA, may offer similar benefits in managing aggression, presenting a potentially safer alternative for individuals concerned about glutamate consumption. Additionally, the activation of the intermediate nucleus of the NTS via the vagus nerve appears to be pivotal in the gut-brain pathway, facilitating the modulation of aggressive behavior by attenuating CeA hyperactivity. Nonetheless, further research is required to elucidate the precise mechanisms underlying these effects in the brain and to identify the specific neural circuits involved in aggression modulation.

## CRediT authorship contribution statement

**Shiori Tominaga:** Validation, Methodology, Investigation. **Shinya Ueno:** Visualization, Validation, Methodology, Investigation. **Dewi Mustika:** Writing – review & editing, Writing – original draft, Validation, Methodology, Investigation, Formal analysis. **Yu Nishimura:** Writing – original draft, Visualization, Validation, Software, Resources, Investigation, Formal analysis, Data curation. **Hideki Hida:** Writing – review & editing, Supervision, Project administration, Funding acquisition, Conceptualization. **Jung ChaGyun:** Validation, Software, Methodology. **Naoki Tajiri:** Validation, Software, Methodology. **Mariko Shindo:** Validation, Software, Methodology, Investigation.

## Declaration of Generative AI and AI-assisted technologies in the writing process

During preparation of this work the authors used Paperpal in order to improve readability and language of the manuscript. After using this tool/service, the author reviewed and edited the content as needed and takes full responsibility for the content of published article

## Declaration of Competing Interest

None.

## Data Availability

The datasets presented in this article are not readily available. Request to access the datasets should be directed to hhida@med.nagoya-cu.ac.jp

## Appendix A Supplementary material

Fig.supplementary Fig. 1 Post-weaning social isolation (PWSI) induces escalation of aggressive behavior in SHR/Izm rats. (A) The frequency of aggressive grooming was significantly higher in the PWSI group compared with the group-housed rats, and attack behaviors—absent under group housing conditions—emerged following PWSI. (B) The duration of aggressive grooming was also significantly increased in the PWSI group, and attack behaviors, which did not occur in group-housed rats, were observed only after PWSI. (C) Latency to aggressive grooming did not differ between groups; however, attack behaviors appeared exclusively in the PWSI group. Data are presented as the mean ± SE (n = 3–4 in each group); **p* < 0.05, ***p* < 0.01, Mann-Whitney test

Fig.supplementary Fig. 2 Daily intake of MSG & MSA in experimental rats and estimation of the human equivalent dose of MSG & MSA. (A) The volume of drinking consumed by SHR/Izm in the control group (dH_2_O; n = 12), MSG group (MSG; n = 12), and MSA group (MSA; n = 12), was measured during five-week PWSI from P25 until P60. (B) Daily intake of MSG and MSA in the MSG group, and MSA group was calculated from the volume of drinking water containing 60 mM MSG and MSA consumed per day. (C) Body weight was measured during five-week PWSI and MSG and MSA administration period from P25 until P60. (D) The dose of MSG and MSA administered was calculated from the daily intake of MSG and MSA per rat body weight. (E) The human equivalent dose (HED) was calculated from the daily intake per kilogram body weight of rats, involving allometric scaling by multiplying with the Km ratio of 0.162, and the adjusted HED was estimated by dividing 20 regarding the high sensitivity of the taste receptor T1R1/T1R3 in humans
