# Supplementary figures and images for: The intake of monosodium aspartate attenuates aggression induced by post-weaning social isolation in an ADHD rat model
Source: J Physiol Sci. 2026 Jan 9;76(1):100056. doi: 10.1016/j.jphyss.2026.100056 (PMC12856302; doi:10.1016/j.jphyss.2026.100056)

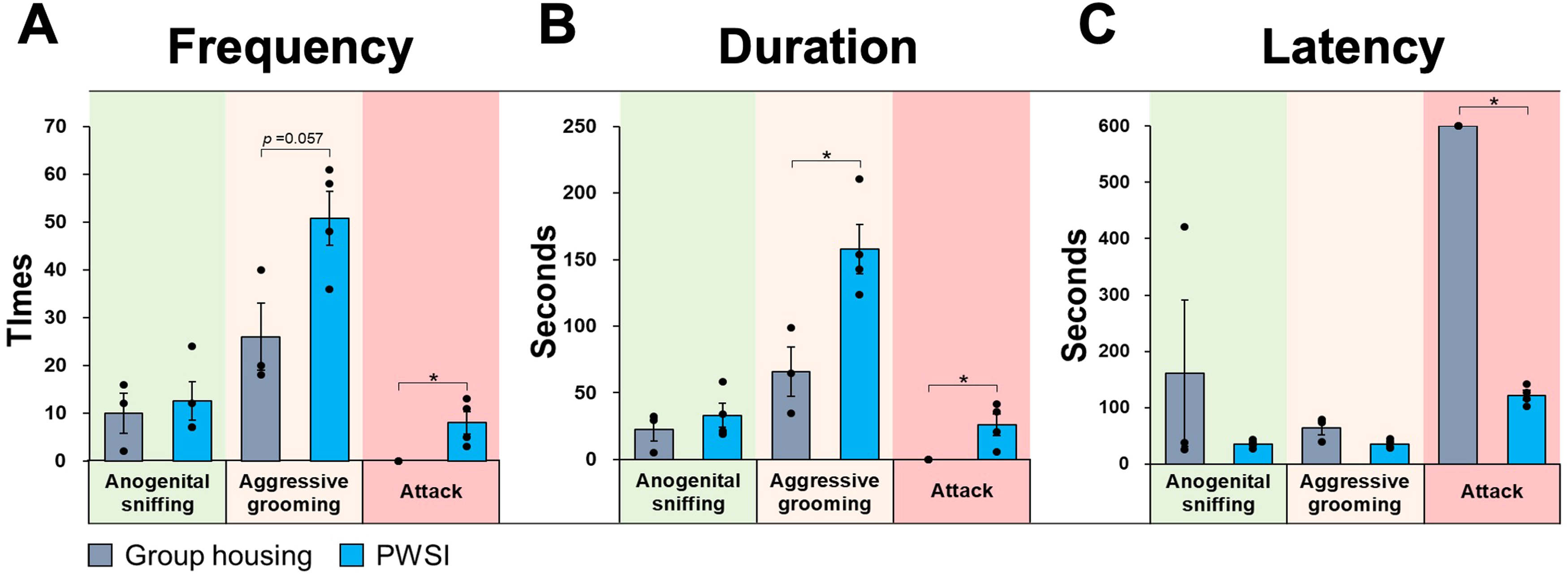

Supplement: Fig.supplementary Fig. 1 — Post-weaning social isolation (PWSI) induces escalation of aggressive behavior in SHR/Izm rats. (A) The frequency of aggressive grooming was significantly higher in the PWSI group compared with the group-housed rats, and attack behaviors—absent under group housing conditions—emerged following PWSI. (B) The duration of aggressive grooming was also significantly increased in the PWSI group, and attack behaviors, which did not occur in group-housed rats, were observed only after PWSI. (C) Latency to aggressive grooming did not differ between groups; however, attack behaviors appeared exclusively in the PWSI group. Data are presented as the mean ± SE (n = 3–4 in each group); *p < 0.05, **p < 0.01, Mann-Whitney test [file mmc1.jpg]

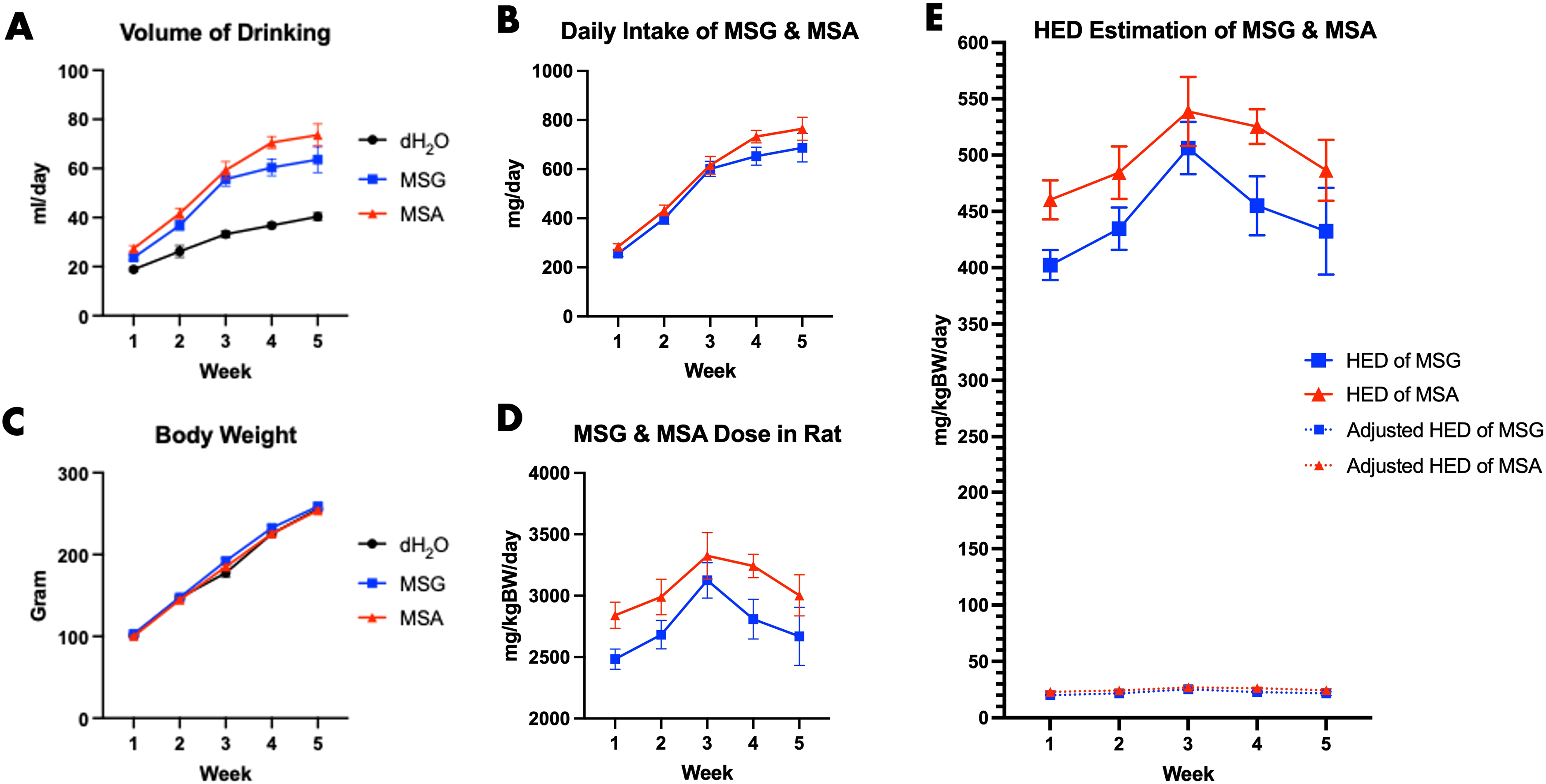

Supplement: Fig.supplementary Fig. 2 — Daily intake of MSG & MSA in experimental rats and estimation of the human equivalent dose of MSG & MSA. (A) The volume of drinking consumed by SHR/Izm in the control group (dH2O; n = 12), MSG group (MSG; n = 12), and MSA group (MSA; n = 12), was measured during five-week PWSI from P25 until P60. (B) Daily intake of MSG and MSA in the MSG group, and MSA group was calculated from the volume of drinking water containing 60 mM MSG and MSA consumed per day. (C) Body weight was measured during five-week PWSI and MSG and MSA administration period from P25 until P60. (D) The dose of MSG and MSA administered was calculated from the daily intake of MSG and MSA per rat body weight. (E) The human equivalent dose (HED) was calculated from the daily intake per kilogram body weight of rats, involving allometric scaling by multiplying with the Km ratio of 0.162, and the adjusted HED was estimated by dividing 20 regarding the high sensitivity of the taste receptor T1R1/T1R3 in humans [file mmc2.jpg]
